# Transcriptome Analysis Reveals a Follicular Microenvironment Melanogenesis Axis in Black-to-White Coat-Color Transition of Junken Meat Sheep

**DOI:** 10.3390/biology15131042

**Published:** 2026-06-30

**Authors:** Binpeng Xi, Sanchuan Zhao, Qian Yu, Huaqian Zhou, Wenzhe Zhang, Yan Chen, Ruiqi Cheng, Zhipeng Wang, Hua Yang, Jianbin Liu

**Affiliations:** 1 State Key Laboratory of Sheep Genetic Improvement and Healthy Production, Xinjiang Academy of Agricultural and Reclamation Science, Shihezi 832000, China; w5562080w@163.com (B.X.); lth112502@163.com (S.Z.); sugeryq@163.com (Q.Y.); 15609939908@163.com (H.Z.); zwz561565@gmail.com (W.Z.); 15739335607@163.com (Y.C.); 18240935520@163.com (R.C.); 2 Key Laboratory of Animal Genetics and Breeding on the Tibetan Plateau, Ministry of Agriculture and Rural Affairs, Lanzhou Institute of Husbandry and Pharmaceutical Sciences, Chinese Academy of Agricultural Sciences, Lanzhou 730050, China; 3 Sheep Breeding Engineering Technology Research Center, Chinese Academy of Agricultural Sciences, Lanzhou 730050, China; 4 College of Animal Science and Technology, Northeast Agricultural University, Haerbin 150030, China; wangzhipeng@neau.edu.cn

**Keywords:** Junken meat sheep, coat-color transition, RNA-seq, melanogenesis, follicular microenvironment

## Abstract

Junken meat sheep exhibit a characteristic postnatal coat-color transition, in which the initially black fleece gradually fades and develops into a white-trunk phenotype; however, the transcriptional basis of this developmental change in follicular pigment output remains unclear. In this study, three Junken meat sheep lambs showing a natural postnatal black-to-white coat-color transition were sampled longitudinally at the newborn black-fleece stage and the 179-day white-trunk stage, generating three matched biological pairs for RNA-seq analysis. Representative candidate genes were further validated by RT-qPCR. Differential expression analysis identified 1657, 400, and 1086 differentially expressed genes in the C11 vs. C1, C22 vs. C2, and C33 vs. C3 comparisons, respectively. Functional enrichment analysis indicated that these genes were mainly associated with tyrosine metabolism, ECM–receptor interaction, focal adhesion, Phosphoinositide 3-kinase-Akt signaling pathway (PI3K-Akt), arachidonic acid metabolism, estrogen signaling, and immune-related pathways. Integrated analysis of shared downregulated genes and expression patterns highlighted candidate genes related to pigmentation, the ECM/follicular microenvironment, and regulatory or metabolic processes. Pigmentation-related genes, including *SOX10*, *TYR*, *TYRP1*, *PMEL*, *OCA2* and *SLC45A2*, were generally downregulated in 179-day white-trunk-stage skin, while changes in ECM- and metabolism-related genes suggested altered follicular microenvironmental regulation. These findings identify candidate transcriptional features associated with developmental coat-color fading in Junken meat sheep and support a follicular microenvironment–melanogenesis expression axis as a transcriptome-based framework for further investigation.

## Simple Summary

Junken meat sheep are born with completely black fleece, but as they grow, the fleece on the body gradually becomes white while the head and limbs remain dark. The biological reason for this developmental color change is still unclear. This study compared skin from black-coated newborn lambs with skin from sheep whose body fleece had turned white after growth, aiming to identify changes in gene activity related to coat-color fading. The results showed that genes involved in pigment production were generally less active in white skin. At the same time, genes related to the local hair follicle environment, tissue support, cell attachment, energy balance, hormone-related regulation, and immune status also changed. These findings suggest that the black-to-white coat-color transition is not caused by a single pigment gene, but may result from coordinated changes in pigment-producing cells and their surrounding hair follicle environment. This study provides useful molecular information for understanding sheep coat-color development and may support future breeding, germplasm evaluation, and studies of local sheep genetic resources.

## 1. Introduction

Coat color is a highly visible trait in sheep and an important indicator for breed identification, germplasm evaluation, and genetic diversity analysis. It also provides valuable insight into breed formation and artificial selection history [1]. As the major tissue responsible for hair growth and pigment deposition, skin provides a biologically relevant system for investigating the molecular processes underlying coat-color formation, wool fiber development, and environmental adaptation [2].

In mammals, coat color is largely determined by the development, maintenance, and functional activity of melanocytes within hair follicles, as well as by melanin synthesis and transport. Melanocytes are derived from neural crest cells and migrate to the epidermis and hair follicles during embryogenesis, where they contribute to pigmentation of the skin and hair shaft [3]. Melanogenesis is not governed by a single enzymatic step; instead, it is a coordinated biological process involving cellular metabolism, redox balance, melanosome maturation, and local microenvironmental cues [4]. Moreover, melanocyte stem cells and their progeny within the hair follicle are required for the cyclic renewal and maintenance of hair pigmentation, and disruption of melanocyte stem cell homeostasis may reduce the capacity for pigment regeneration [5]. Accordingly, coat-color variation should be considered not merely as the result of altered pigmentation-gene expression, but as a composite phenotype shaped by melanocyte developmental status, melanogenic capacity, and the follicular microenvironment.

The hair follicle is a dynamic niche composed of epithelial, mesenchymal, pigment, vascular, and immune cells, together with the extracellular matrix. Beyond providing structural support, the extracellular matrix can regulate melanocyte behavior through cell adhesion, Mechan sensing, and local signaling [6]. In sheep, pathways such as ECM–receptor interaction, focal adhesion, and estrogen signaling have been linked to hair follicle morphogenesis and wool fiber development [7]. Multi-omics studies of black- and white-coated sheep have further revealed molecular differences associated with pigmentation, local metabolism, and tissue homeostasis [8,9]. These findings suggest that coat color formation is not solely governed by classical melanogenesis but is also closely associated with the follicular microenvironment.

Current research on sheep coat color has largely focused on cross-sectional comparisons among stable coat-color phenotypes, including black, white, or individuals with atypical melanin deposition. Integrated genomic and transcriptomic analyses have been used to characterize the genetic basis of high melanin deposition in black-boned sheep and black–white coat-color differences in Tibetan sheep [10,11]. However, the gradual postnatal transition from black to white represents a dynamic developmental phenotype that is distinct from static differences between black and white individuals. Hair pigmentation loss has been associated with alterations in melanocyte stem cell status, changes in the follicular microenvironment, oxidative damage, and functional decline of the hair follicle pigmentary unit [12,13]. Moreover, studies of m6A methylation and m6A-modified lncRNAs suggest that epitranscriptomic regulation and non-coding RNAs may also contribute to pigmentation processes in sheep skin [14,15]. Therefore, comparisons based only on fixed coat-color phenotypes or a small number of classical pigmentation genes are insufficient to capture the tissue-level transcriptional changes underlying developmental coat-color transition.

Junken meat sheep are a newly developed meat-type sheep germplasm resource in Xinjiang, with favorable environmental adaptability, reproductive performance, and meat production potential. Individuals of this breed are born with an entirely black fleece; during postnatal development, the fleece on the trunk progressively fades and turns white, whereas the head and limbs largely retain black pigmentation, forming a breed-associated developmental black-to-white coat-color transition phenotype. Unlike conventional comparisons between black and white individuals, this naturally occurring color change reflects a continuous developmental process. Junken meat sheep therefore provide a valuable model for investigating pigmentation attenuation, follicular microenvironmental changes, and transcriptional regulation during developmental coat-color transition in sheep.

Based on this background, the present study used black skin and developmentally whitened skin from Junken meat sheep to perform comparative transcriptomic analysis by RNA-seq, with representative candidate genes further validated by RT-qPCR. This study aimed to systematically characterize transcriptional changes and potential functional processes associated with the black-to-white coat-color transition in skin tissue. By integrating differential expression analysis, functional enrichment, and candidate gene expression-pattern screening, this work sought to identify key transcriptional features underlying developmental coat-color transition in Junken meat sheep, thereby providing molecular evidence for understanding pigmentation attenuation in sheep and theoretical support for the evaluation and utilization of local sheep germplasm resources.

## 2. Materials and Methods

### 2.1. Ethics Statement

All experimental procedures were conducted in accordance with the relevant guidelines for the care and use of experimental animals and were approved by the Experimental Animal Management and Use Committee of the Xinjiang Academy of Agricultural and Reclamation Sciences, Shihezi, China. The ethical approval number was XJNKKXY-2020-34, and the approval date was 30 December 2020. During animal handling and sample collection, all procedures were performed under veterinary supervision, and every effort was made to minimize pain, stress, and discomfort.

### 2.2. Experimental Animals and Sample Collection

Junken meat sheep used in this study were obtained from the sheep breeding farm of the Xinjiang Academy of Agricultural and Reclamation Sciences. A longitudinal sampling design was used to investigate transcriptomic changes associated with the postnatal black-to-white coat-color transition. Three Junken meat sheep lambs with an entirely black fleece at birth were selected and followed during postnatal development. At the newborn stage, skin biopsy samples were collected from the standardized dorsal region and designated C1, C2, and C3. The same individuals were sampled again at 179 days of age, when the fleece on the trunk had developmentally turned white, and the corresponding samples were designated C11, C22, and C33, respectively. Therefore, C11, C22, and C33 represent the 179-day developmental samples corresponding to C1, C2, and C3 from the same individuals.

No animals were euthanized or sacrificed for sample collection. All skin samples were collected by live skin biopsy under veterinary supervision, and the sampling site was standardized to the same dorsal region. Before biopsy, animals were gently restrained, the fleece over the sampling area was clipped, and the skin was disinfected sequentially with povidone-iodine and 75% ethanol. Local infiltration anesthesia was performed using 1% lidocaine hydrochloride at 3 mg/kg body weight, with the total dose not exceeding 6 mg/kg body weight. After the absence of a local pain response was confirmed, full-thickness skin tissue was collected using sterile surgical instruments. Following biopsy, hemostasis was achieved by compression, the wound was closed with simple interrupted sutures, and the surgical site was disinfected. Meloxicam was administered for postoperative analgesia at 0.5 mg/kg body weight. Skin tissues were immediately snap-frozen in liquid nitrogen and stored at −80 °C until RNA extraction. Animals were monitored routinely after sampling, and no obvious postoperative complications were observed. To improve clarity and standardize sample terminology, detailed sample information is provided in Supplementary Table S1. Throughout the manuscript, C1, C2, and C3 denote samples from the newborn black-fleece stage, whereas C11, C22, and C33 denote samples from the same individuals at the 179-day white-trunk stage. Accordingly, C11 vs. C1, C22 vs. C2, and C33 vs. C3 represent matched longitudinal comparisons.

### 2.3. RNA Extraction and Quality Assessment

Total RNA was extracted from skin tissues using the SteadyPure Universal RNA Extraction Kit (Tiangen, Beijing, China) according to the manufacturer’s instructions. RNA concentration and purity were assessed using a NanoDrop 2000 spectrophotometer (Thermo Fisher Scientific, Waltham, MA, USA), and RNA integrity was evaluated using an Agilent Bioanalyzer 2100 system (Agilent Technologies, Santa Clara, CA, USA). Only RNA samples with an RNA integrity number (RIN) greater than 8.0 were used for subsequent library construction and sequencing analysis.

### 2.4. Library Construction and Sequencing

After quality assessment of total RNA, mRNA was enriched using oligo(dT) beads and subsequently fragmented. First-strand cDNA was synthesized from the fragmented mRNA using random primers, followed by second-strand cDNA synthesis with DNA polymerase I, RNase H, dNTPs, and the appropriate reaction buffer. The resulting double-stranded cDNA was purified using the QIAquick PCR Purification Kit (QIAGEN, Venlo, The Netherlands), followed by end repair, A-tailing, and ligation of Illumina sequencing adapters.

Sequencing libraries were constructed and sequenced by Novogene Co., Ltd. (Guangzhou, China) on an Illumina HiSeq 2500 platform to generate paired-end reads. Raw reads were subjected to quality control to remove adapter-contaminated reads, low-quality reads, and reads containing excessive undetermined bases. The resulting high-quality clean reads were mapped to the sheep reference genome Oar_v4.0 (NCBI: GCF_000298735.2) using HISAT2. The raw RNA-seq data have been submitted to the NCBI Sequence Read Archive (SRA) under the BioProject accession number PRJNA1462536 (https://www.ncbi.nlm.nih.gov/bioproject/PRJNA1462536, accessed on 6 May 2026).

### 2.5. Differential Expression Analysis

Gene-level read counts were generated from the mapped reads using featureCounts (Subread 2.1.1). Genes with no read counts across all samples were removed before differential expression analysis. Differential expression analysis was performed using edgeR (edgeR 4.10.1)based on gene-level raw count data. Raw counts were normalized using the trimmed mean of M values (TMM) method to correct for differences in sequencing depth and library composition among samples. Differential expression was assessed using a negative binomial model, and *p* values were adjusted for multiple testing using the Benjamini–Hochberg method.

Three matched individual comparisons were analyzed: C11 vs. C1, C22 vs. C2, and C33 vs. C3. In these comparisons, C11, C22, and C33 represented 179-day white-trunk-stage samples from the same individuals sampled at birth as C1, C2, and C3, respectively. Genes with |log2 fold change| ≥ 1.0 and adjusted *p* value (padj) ≤ 0.05 were considered differentially expressed.

The resulting DEG sets were used to characterize within-individual transcriptional changes during the natural developmental black-to-white coat-color transition. Genes and pathways associated with pigmentation, the follicular microenvironment, and regulatory or metabolic processes were prioritized for downstream analyses. FPKM and log2(FPKM + 1)-transformed expression values were used only for expression visualization and heatmap analysis, not for differential expression testing.

### 2.6. Functional Enrichment Analysis of Differentially Expressed Genes

Gene Ontology (GO) enrichment and Kyoto Encyclopedia of Genes and Genomes (KEGG) pathway analyses of the differentially expressed gene sets were conducted using the clusterProfiler packagev4.20.0 in R v4.6.0. Statistical significance was evaluated based on a hypergeometric distribution test, with the entire set of analyzed genes used as the background. GO and KEGG enrichment analyses were performed using the clusterProfiler packagev4.20.0 in R v4.6.0. *p* values were adjusted using the Benjamini–Hochberg method, and terms or pathways with adjusted *p* value (padj) ≤ 0.05 were considered significantly enriched.

### 2.7. Reverse Transcription Quantitative PCR Validation

To validate the RNA-seq results, 12 differentially expressed genes were selected for RT-qPCR analysis. Primer sequences are listed in Supplementary Table S2. RT-qPCR was performed using the same six RNA samples as the RNA-seq analysis, including three matched biological pairs (C1/C11, C2/C22, and C3/C33). Total RNA was reverse-transcribed into cDNA using the RevertAid First Strand cDNA Synthesis Kit (Thermo Fisher Scientific, Waltham, MA, USA), and RT-qPCR was conducted using the QuantiNova SYBR Green PCR Kit (QIAGEN, Shanghai, China). GAPDH was used as the reference gene, and relative expression levels were calculated using the 2^−ΔΔCt^ method. Each sample was analyzed with three technical replicates. RT-qPCR was used to verify the consistency of expression trends observed in the RNA-seq data.

### 2.8. Statistical Analysis

All data are presented as the mean ± standard error of the mean (SEM). Statistical analyses were performed using GraphPad Prism 8 software. Comparisons between two groups were conducted using Student’s *t*-test, whereas comparisons among multiple groups were analyzed by one-way analysis of variance (ANOVA) followed by Bonferroni’s multiple-comparison test. A value of *p* ≤ 0.05 was considered statistically significant.

## 3. Results

### 3.1. Gene Expression Profile Analysis During the Black-to-White Coat-Color Transition in Junken Meat Sheep

Six cDNA libraries were constructed from six skin RNA samples, followed by quality control and statistical assessment of the RNA-seq data. In total, 270.56 million raw reads were generated from the six libraries, and 263.99 million clean reads were retained after quality filtering (Supplementary Table S2). The sequencing error rate was 0.03% for all samples, with Q20 values ranging from 97.57% to 97.76% and Q30 values ranging from 93.19% to 93.76%, indicating that the sequencing data were of sufficient quality for subsequent transcriptomic analysis. The GC content of the six samples ranged from 45.51% to 51.50%, showing a stable overall distribution and no obvious bias in library base composition (Supplementary Table S3).

After mapping the clean reads to the reference genome, the total mapping rate ranged from 87.01% to 89.09% across samples. The uniquely mapped read rate ranged from 80.06% to 82.23%, whereas the multiple-mapping rate ranged from 5.43% to 8.41%, indicating that most reads were effectively aligned to the sheep reference genome (Supplementary Table S4). Further analysis of genomic region distribution showed that 71.65–82.50% of the mapped reads were located in exonic regions, 6.06–15.00% in intronic regions, and 10.94–13.35% in intergenic regions. These results indicate that the transcriptomic data had good annotation coverage and were suitable for downstream analysis (Supplementary Table S5).

### 3.2. Comparative Analysis of Differentially Expressed Genes Between Black and White Skin and Identification of Shared Key Genes

**Figure 1:**
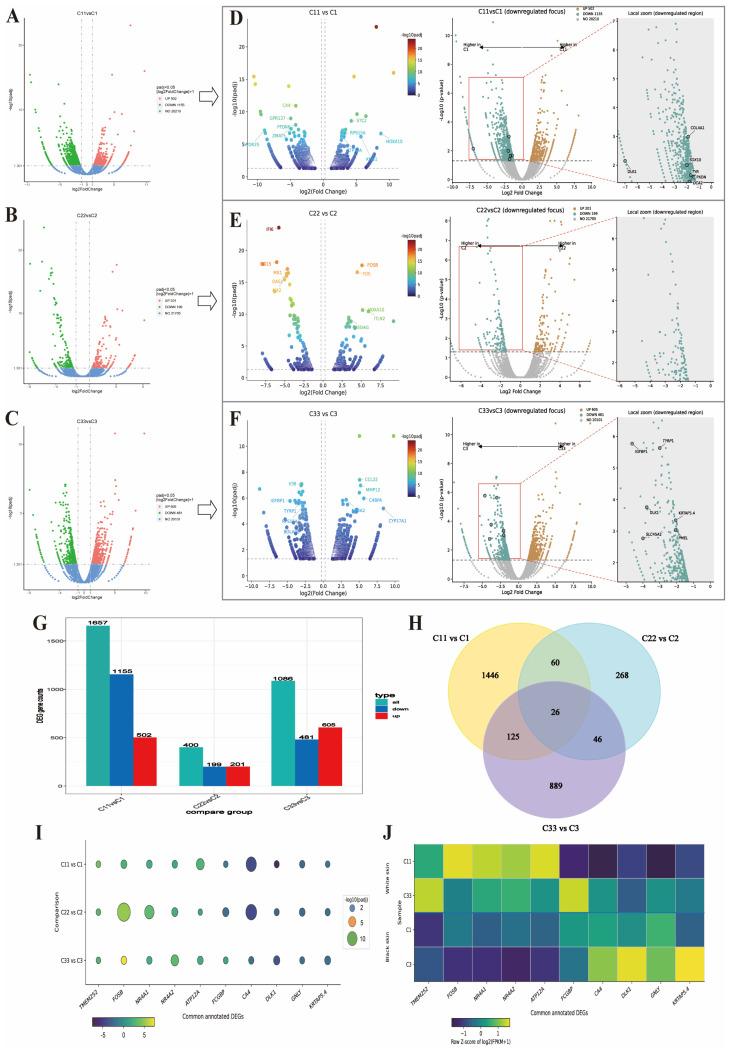
Comparative analysis of differentially expressed genes between black and white skin and identification of shared key genes. (**A**–**C**) Volcano plots of DEGs for C11 vs. C1, C22 vs. C2, and C33 vs. C3, respectively, showing the overall distribution of DEGs in each comparison. The *x*-axis indicates log2FoldChange, and the *y*-axis indicates −log10 (padj). Red and green dots represent significantly upregulated and downregulated genes, respectively, while gray dots indicate non-significant genes. (**D**–**F**) Highlighted DEGs and local enlarged views of the downregulated regions for C11 vs. C1, C22 vs. C2, and C33 vs. C3, respectively. The middle panels highlight representative DEGs, and the right panels provide enlarged views of the downregulated regions to show candidate genes potentially associated with coat color change. (**G**) Statistics of the total number of DEGs and the numbers of upregulated and downregulated genes in the three comparisons. (**H**) Venn diagram showing the overlap of DEGs among the three comparisons, including comparison-specific and shared DEGs. (**I**) Bubble heatmap of shared annotated key genes across the three comparisons. The *x*-axis represents the shared key genes and the *y*-axis represents the comparisons. Bubble color indicates log2FoldChange, and bubble size indicates the significance level [−log10(padj)]. (**J**) Expression heatmap of shared key genes in individual samples. The heatmap was generated using samples after removal of the second replicate group (C11, C33, C1, and C3). Colors represent the relative expression levels after log2(FPKM + 1) transformation and row-wise normalization.

To investigate transcriptional changes in skin tissue during the black-to-white coat-color transition in Junken meat sheep, differential expression analysis was performed between black skin and developmentally whitened skin samples. Significant differentially expressed genes were detected in all three comparisons; however, the magnitude and direction of transcriptional changes were not completely consistent among the paired comparisons, suggesting a certain degree of individual variation or local tissue-level transcriptional heterogeneity during coat-color transition (Figure 1A–G). Specifically, 1657, 400, and 1086 differentially expressed genes were identified in the C11 vs. C1, C22 vs. C2, and C33 vs. C3 comparisons, respectively. Overall, downregulated genes predominated in C11 vs. C1, whereas upregulated genes predominated in C33 vs. C3. By contrast, C22 vs. C2 contained the fewest differentially expressed genes and showed a relatively limited overall transcriptional response.

Because this study focused on candidate transcriptional changes consistent with the biological process of black-to-white coat-color transition, the downregulated regions in the three comparisons were further examined using locally enlarged volcano plots. In both C11 vs. C1 and C33 vs. C3, a set of representative downregulated genes related to pigmentation, follicular structure, and local microenvironmental regulation was observed, including *SOX10*, *TYR*, *PXDN*, *COL4A1*, *TYRP1*, *PMEL*, *SLC45A2* and *DLK1*. In contrast, the enlarged region of C22 vs. C2 did not show typical pigmentation- or follicular microenvironment-related genes directly associated with coat-color change. Although all samples met RNA-seq quality standards, C22 vs. C2 yielded the fewest DEGs and showed no clear pigmentation-related signals. The weaker transcriptional differences may reflect biological variation or local tissue heterogeneity. Therefore, C22 vs. C2 was included in the overall DEG analysis, while candidate gene screening focused mainly on C11 vs. C1 and C33 vs. C3. Instead, the differential signals in this comparison were mainly represented by response-related genes, including *IFI6*, *ISG15*, *MX1*, *OAS1*, *FOS*, and *FOSB*, indicating that coat-color-associated transcriptional features were less pronounced in this comparison.

Venn analysis further identified 26 shared differentially expressed genes across the three comparisons (Figure 1H). A bubble plot comparing the annotated shared genes showed that their expression trends were not fully consistent among the three comparisons, with particularly large fluctuations in the second comparison, suggesting relatively weak overall stability (Figure 1I). Therefore, the first and third comparisons, which showed clearer differential signals, were selected for further heatmap analysis (Figure 1J). The results showed that *TMEM252*, *FOSB*, *NR4A1*, *NR4A2*, and *ATP12A* were relatively highly expressed in white skin samples, whereas *CA4*, *DLK1*, *GNLY*, and *KRTAP5.4* were relatively highly expressed in black skin samples. Overall, these shared differentially expressed genes exhibited more consistent expression directions in the first and third comparisons and were partially consistent with transcriptional changes associated with the black-to-white coat-color transition, suggesting that the shared differential signals extracted from the three comparisons have potential biological relevance.

### 3.3. GO and KEGG Enrichment Analyses Reveal Distinct Functional Patterns of Differentially Expressed Genes Between Black and White Skin

**Figure 2:**
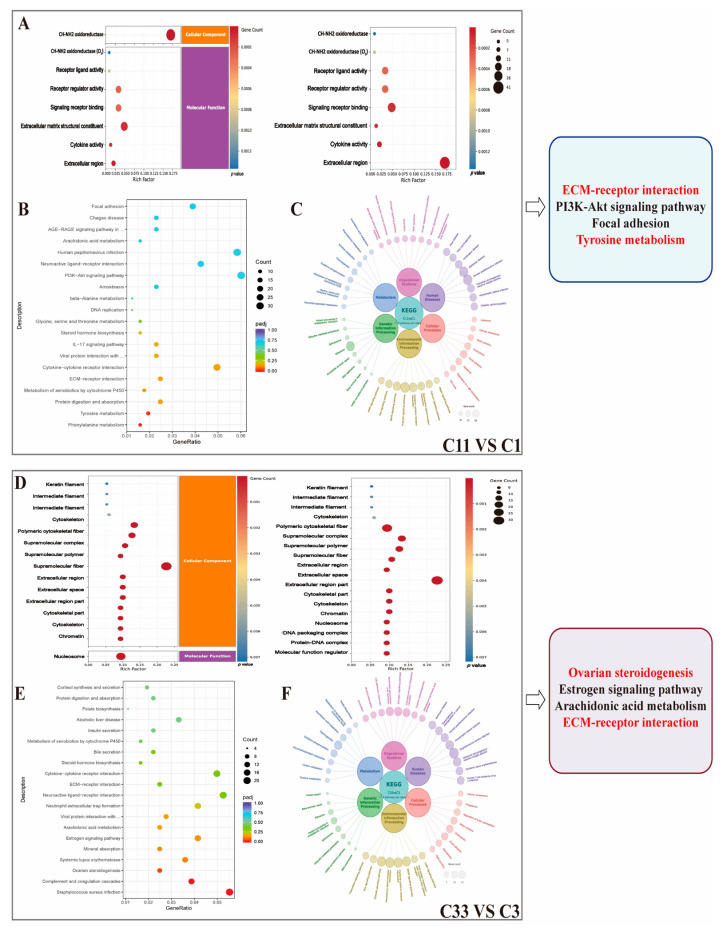
Functional enrichment analysis of differentially expressed genes in C11 vs. C1 and C33 vs. C3. (**A**) GO enrichment analysis of differentially expressed genes in C11 vs. C1. (**B**) KEGG enrichment analysis of differentially expressed genes in C11 vs. C1. (**C**) KEGG hierarchical classification of significantly enriched pathways in C11 vs. C1. (**D**) GO enrichment analysis of differentially expressed genes in C33 vs. C3. (**E**) KEGG enrichment analysis of differentially expressed genes in C33 vs. C3. (**F**) KEGG hierarchical classification of significantly enriched pathways in C33 vs. C3. In the bubble plots, bubble size represents gene count and color indicates the adjusted *p* value. The KEGG hierarchical plots show the distribution of significantly enriched pathways across major functional categories.

To further clarify the potential biological functions of differentially expressed genes between black and white skin, GO and KEGG enrichment analyses were performed separately for the C11 vs. C1 and C33 vs. C3 comparisons. KEGG pathway classification was further used to summarize the functional categories of significantly enriched pathways (Figure 2). Overall, the differentially expressed genes in both comparisons were mainly associated with changes in the extracellular microenvironment, receptor-ligand interactions, metabolic regulation, and cellular processes, although the enrichment patterns differed between comparisons.

In the C11 vs. C1 comparison, the enriched GO terms were mainly associated with extracellular region, extracellular matrix structural constituent, cytokine activity, receptor ligand activity, receptor regulator activity, signaling receptor binding, and oxidoreductase activity acting on CH-NH2 group donors. These results indicated that the differentially expressed genes in this comparison were primarily related to extracellular-region functions, ligand-receptor signaling, and changes in oxidoreductase-related enzymatic activity (Figure 2A). KEGG enrichment analysis further showed that these genes were significantly enriched in pathways such as phenylalanine metabolism, tyrosine metabolism, ECM–receptor interaction, cytokine–cytokine receptor interaction, IL-17 signaling pathway, PI3K-Akt signaling pathway, focal adhesion, and arachidonic acid metabolism. Among these pathways, tyrosine metabolism and phenylalanine metabolism are closely related to substrate metabolism for pigment synthesis, whereas enrichment of ECM–receptor interaction, focal adhesion, and PI3K-Akt signaling suggests changes in perifollicular matrix signaling and cell adhesion-related processes. In addition, enrichment of cytokine–cytokine receptor interaction, IL-17 signaling, and arachidonic acid metabolism indicated that local cytokine signaling, inflammatory responses, and lipid-mediated regulation may also be associated with differences between black and white skin (Figure 2B). KEGG pathway classification showed that the significant pathways in this comparison were mainly assigned to metabolism, environmental information processing, cellular processes, and organismal systems, suggesting that the C11 vs. C1 comparison was characterized primarily by changes in metabolic and signaling-regulatory processes (Figure 2C).

In the C33 vs. C3 comparison, GO enrichment highlighted more structure-related terms, including keratin filament, intermediate filament, cytoskeleton, polymeric cytoskeletal fiber, supramolecular complex, extracellular region, extracellular space, chromatin, nucleosome, DNA packaging complex, and protein-DNA complex. These results suggested that the differentially expressed genes in this comparison were mainly associated with follicular or epidermal structural components, cytoskeletal organization, and changes in nuclear chromatin packaging status (Figure 2D). KEGG enrichment analysis showed that the differentially expressed genes were significantly enriched in complement and coagulation cascades, ovarian steroidogenesis, estrogen signaling pathway, arachidonic acid metabolism, ECM–receptor interaction, cytokine–cytokine receptor interaction, neuroactive ligand–receptor interaction, and neutrophil extracellular trap formation. These pathways indicated that, in addition to changes in extracellular matrix and cytokine signaling, this comparison also involved more pronounced hormone-related metabolic and immune-response processes (Figure 2E). KEGG pathway classification similarly showed that the significant pathways were mainly distributed across metabolism, environmental information processing, cellular processes, and organismal systems. Compared with C11 vs. C1, however, C33 vs. C3 displayed stronger features related to structural components, the tissue environment, and immune or hormone-associated processes (Figure 2F).

Overall, enrichment results from both comparisons indicated that the black-to-white coat-color transition was accompanied by changes in the extracellular microenvironment, cell communication, and metabolic state. However, C11 vs. C1 was more closely associated with pigmentation-related metabolism and receptor signaling, whereas C33 vs. C3 was more strongly linked to follicular structural components, cytoskeletal organization, and immune or hormone-related changes. These findings suggest that the black-to-white coat-color transition in Junken meat sheep is unlikely to be explained by a single pigmentation pathway alone, but may instead involve coordinated changes in pigment metabolism, the follicular microenvironment, and tissue structural status.

### 3.4. Downregulated Genes Further Define Suppressed Functional Modules Associated with Coat-Color Fading

GO and KEGG enrichment analyses of all differentially expressed genes indicated that both the C11 vs. C1 and C33 vs. C3 comparisons involved changes in the extracellular microenvironment, metabolic regulation, cell communication, and tissue structure. Because the black-to-white coat-color transition is expected to be accompanied by reduced melanogenic capacity, decreased pigment-cell activity, and attenuation of follicular supportive signals, we further focused on downregulated genes to identify functional modules more consistent with coat-color fading.

**Figure 3:**
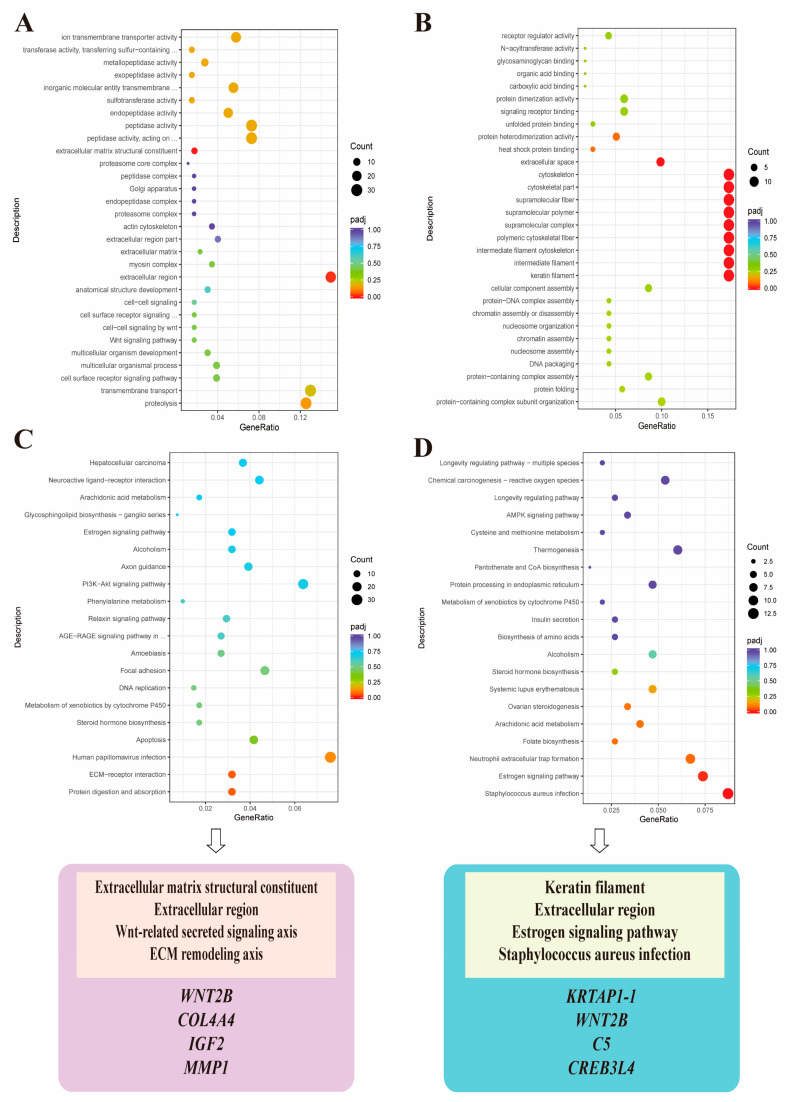
GO and KEGG enrichment analyses of downregulated genes in the C11 vs. C1 and C33 vs. C3 comparisons. (**A**) GO enrichment analysis of downregulated genes in the C11 vs. C1 comparison. (**B**) KEGG pathway enrichment analysis of downregulated genes in the C11 vs. C1 comparison. (**C**) GO enrichment analysis of downregulated genes in the C33 vs. C3 comparison. (**D**) KEGG pathway enrichment analysis of downregulated genes in the C33 vs. C3 comparison. In the bubble plots, the *x*-axis represents the Rich factor or GeneRatio, and the *y*-axis represents enriched GO terms or KEGG pathways. Bubble size indicates the number of enriched genes, while bubble color represents the enrichment significance level, as indicated by the corresponding color scale.

In C11 vs. C1, downregulated genes were mainly enriched in GO terms related to the extracellular region, extracellular matrix structural constituent, receptor ligand activity, receptor regulator activity, signaling receptor binding, cytokine activity, and oxidoreductase activity acting on CH-NH2 group donors (Figure 3A). KEGG analysis further showed enrichment in ECM–receptor interaction, PI3K-Akt signaling pathway, focal adhesion, and tyrosine metabolism (Figure 3C). The enrichment of tyrosine metabolism is consistent with reduced substrate metabolism for melanin synthesis, whereas the enrichment of ECM–receptor interaction, focal adhesion, and PI3K-Akt signaling suggests attenuation of perifollicular matrix interactions, cell adhesion, and cell survival/proliferation-related signals. These results indicate that downregulated genes in C11 vs. C1 mainly reflect reduced pigment-substrate metabolism and weakened follicular microenvironmental support. In C33 vs. C3, downregulated genes exhibited more prominent structural features, with GO enrichment mainly involving keratin filament, intermediate filament, cytoskeleton, polymeric cytoskeletal fiber, supramolecular complex, extracellular region, and extracellular space (Figure 3B). These terms suggest changes in follicular or epidermal structural components, cytoskeletal organization, and local extracellular support. KEGG enrichment further highlighted ovarian steroidogenesis, estrogen signaling pathway, arachidonic acid metabolism, and ECM–receptor interaction (Figure 3D), indicating that hormone-related metabolism, estrogen signaling, lipid-mediated regulation, and ECM-associated signals may also participate in the local follicular microenvironmental changes accompanying coat-color fading.

### 3.5. Module Analysis of Shared Downregulated Genes Identifies Candidate Functional Modules Associated with Coat-Color Fading

**Figure 4:**
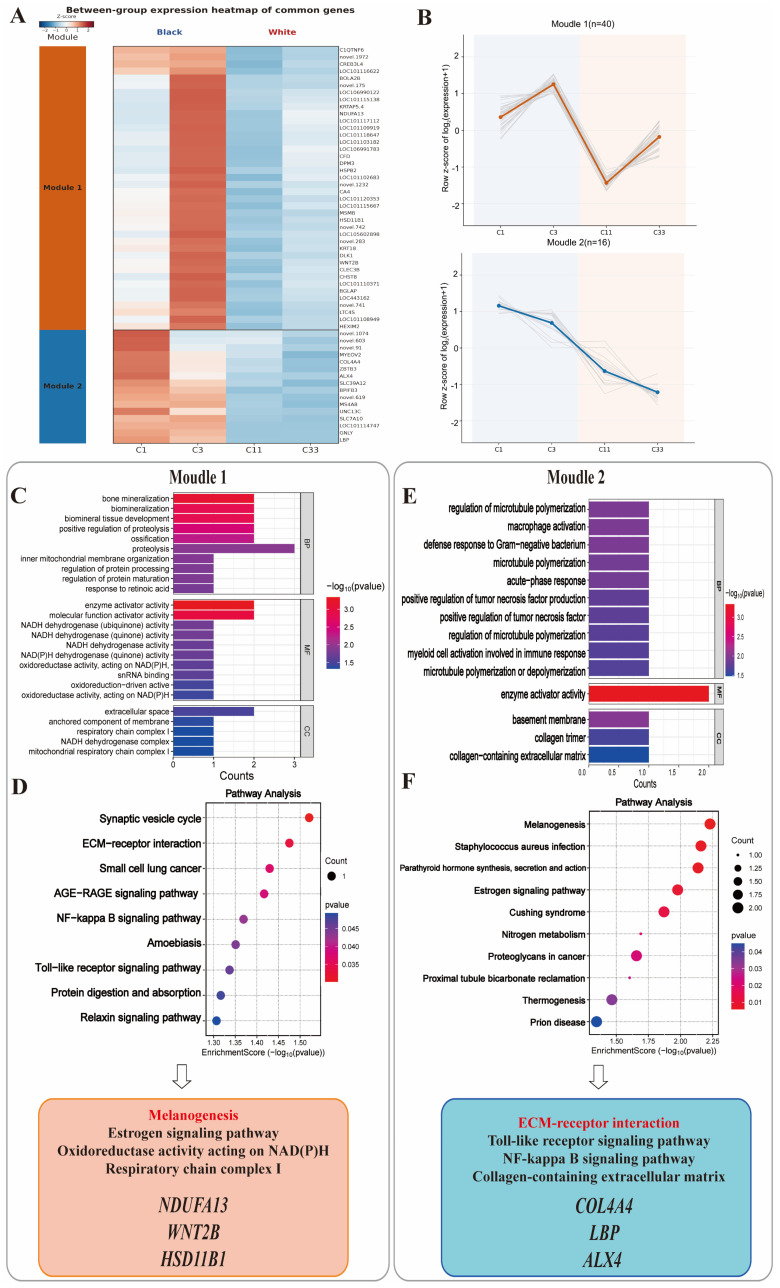
Module analysis and functional enrichment of commonly downregulated genes in the C11 vs. C1 and C33 vs. C3 comparisons. (**A**) Heatmap showing the expression patterns of commonly downregulated genes in black and white skin samples. Genes were clustered into two major modules based on their expression profiles. (**B**) Expression trends of Module 1 and Module 2 across C1, C3, C11, and C33 samples. The *y*-axis represents the row-scaled log2-transformed expression values. (**C**) GO enrichment analysis of genes in Module 1. (**D**) KEGG pathway enrichment analysis of genes in Module 1, with selected candidate pathways and genes highlighted below. (**E**) GO enrichment analysis of genes in Module 2. (**F**) KEGG pathway enrichment analysis of genes in Module 2.

Because downregulated genes in the C11 vs. C1 and C33 vs. C3 comparisons were closely aligned with pigmentation attenuation, follicular microenvironmental changes, and tissue structural remodeling, shared downregulated genes between these two comparisons were further subjected to module analysis (Figure 4). Clustering analysis divided these genes into two major expression modules: Module 1, containing 40 genes, and Module 2, containing 16 genes. Both modules showed clear separation between black and white skin samples, with higher expression in black skin and reduced expression in white skin. Module 1 showed high expression in black skin, particularly in C3, and was markedly reduced in C11 and C33, whereas Module 2 displayed a more continuous decline from black to white skin. These expression patterns suggest that the two modules may represent suppressed functional units associated with coat-color fading (Figure 4A,B).

GO enrichment analysis showed that Module 1 was mainly associated with biomineralization, positive regulation of proteolysis, proteolysis, response to retinoic acid, oxidoreductase activity acting on NAD(P)H, NADH dehydrogenase activity, and respiratory chain complex I-related terms (Figure 4C). KEGG analysis linked this module to ECM–receptor interaction, NF-kappa B signaling pathway, Toll-like receptor signaling pathway, and AGE–RAGE signaling pathway (Figure 4D). Considering the biological context of pigmentation loss, melanogenesis, estrogen signaling pathway, redox-related activity, and mitochondrial respiratory chain function were prioritized as key functional directions, with *NDUFA13*, *WNT2B*, and *HSD11B1* selected as representative candidate genes. Thus, Module 1 may reflect coordinated attenuation of pigmentation-related regulation, energy metabolism, redox homeostasis, and hormone-associated signaling.

Module 2 was enriched in GO terms related to regulation of microtubule polymerization, microtubule polymerization, macrophage activation, acute-phase response, basement membrane, collagen trimer, and collagen-containing extracellular matrix (Figure 4E), indicating stronger associations with cytoskeletal regulation, collagen-related ECM components, basement membrane structure, and immune-inflammatory processes. KEGG analysis further highlighted melanogenesis, estrogen signaling pathway, Staphylococcus aureus infection, thermogenesis, ECM–receptor interaction, Toll-like receptor signaling pathway, and NF-kappa B signaling pathway (Figure 4F). Based on its expression pattern and functional relevance, ECM–receptor interaction, Toll-like receptor signaling pathway, NF-kappa B signaling pathway, and collagen-containing extracellular matrix were prioritized, with *COL4A4*, *LBP*, and *ALX4* selected as representative candidate genes. These findings suggest that Module 2 may capture changes in follicular ECM/collagen support, basement membrane stability, and immune-inflammatory microenvironmental signals during coat-color fading.

### 3.6. Integrated Prioritization of Candidate Genes Associated with the Black-to-White Coat-Color Transition

**Figure 5:**
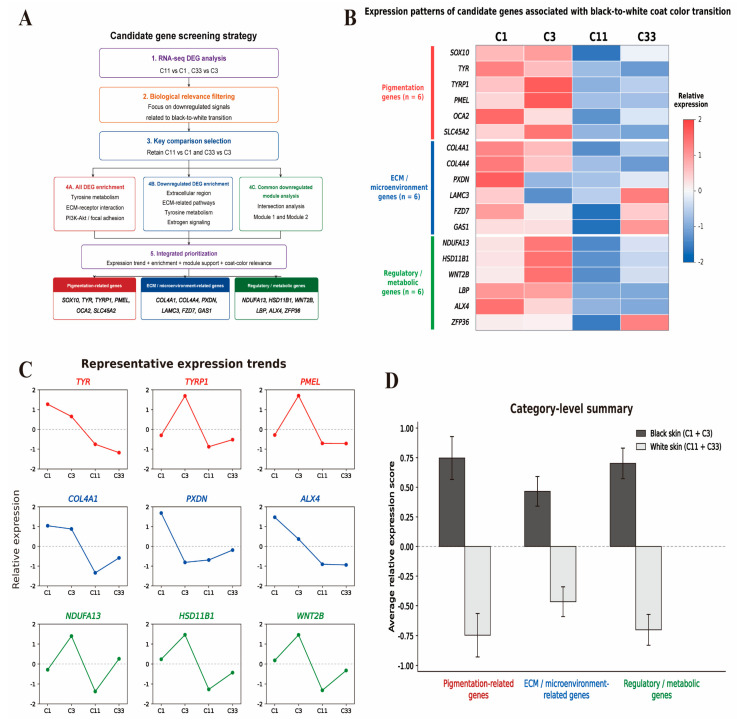
Integrated prioritization and expression profiling of candidate genes associated with black-to-white coat color transition. (**A**) Candidate gene screening strategy based on RNA-seq DEG analysis, biological relevance filtering, key comparison selection, functional enrichment, downregulated DEG enrichment, and common downregulated gene module analysis. Candidate genes were prioritized by integrating expression trends, enrichment results, module support, and coat-color relevance, and were classified into pigmentation-related genes, ECM/microenvironment-related genes, and regulatory/metabolic genes. (**B**) Heatmap showing the relative expression patterns of prioritized candidate genes across C1, C3, C11, and C33. Candidate genes were grouped into three functional categories. Expression values were normalized by row-wise z-score. Red indicates higher relative expression, whereas blue indicates lower relative expression. (**C**) Representative expression trends of selected candidate genes from the three functional categories. Pigmentation-related genes are shown in red, ECM/microenvironment-related genes in blue, and regulatory/metabolic genes in green. The horizontal dashed line indicates a relative expression value of zero. (**D**) Category-level summary of average relative expression scores in black skin samples and white skin samples. Black skin values represent the average relative expression scores of C1 and C3, while white skin values represent those of C11 and C33. Error bars indicate variation among genes within each category.

To prioritize candidate genes associated with the black-to-white coat-color transition, we integrated differential expression patterns, enrichment results of downregulated genes, shared downregulated gene modules, functional annotations, and biological relevance to pigmentation. Candidate genes were grouped into three functional categories: pigmentation-related genes, ECM/follicular microenvironment-related genes, and regulatory/metabolic genes (Figure 5A).

The candidate gene heatmap showed clear expression separation between black and white skin samples across these three categories (Figure 5). Pigmentation-related genes, including *SOX10*, *TYR*, *TYRP1*, *PMEL*, *OCA2* and *SLC45A2*, were expressed at higher levels in black skin and reduced in white skin, suggesting attenuation of melanocyte lineage maintenance, melanin synthesis, and melanosome formation. ECM/follicular microenvironment-related genes, including *COL4A1*, *COL4A4*, *PXDN*, *LAMC3*, *FZD7* and *GAS1*, also showed decreased expression, indicating possible changes in extracellular matrix organization, basement membrane structure, and the local follicular supportive niche. Regulatory/metabolic genes, including *NDUFA13*, *HSD11B1*, *WNT2B*, *LBP*, *ALX4* and *ZFP36*, further pointed to potential involvement of redox metabolism, hormone signaling, inflammatory responses, and transcriptional regulation. Representative gene trend analysis further supported these patterns: *TYR*, *TYRP1* and *PMEL* were reduced in white skin, consistent with weakened melanogenesis-related transcriptional activity; *COL4A1*, *PXDN* and *FZD7* showed decreasing trends, suggesting altered follicular ECM and cell-adhesion-related microenvironmental signals; and *NDUFA13*, *HSD11B1* and *WNT2B* reflected possible changes in metabolic and local regulatory pathways (Figure 5C). At the category level, all three candidate gene groups showed higher average relative expression in black skin than in white skin (Figure 5D). These findings suggest that coat-color fading in Junken meat sheep is associated not only with reduced melanogenic gene expression but also with coordinated attenuation of follicular microenvironmental support and regulatory/metabolic activity.

### 3.7. Transcriptome-Supported Expression Model of the Black-to-White Coat-Color Transition

**Figure 6:**
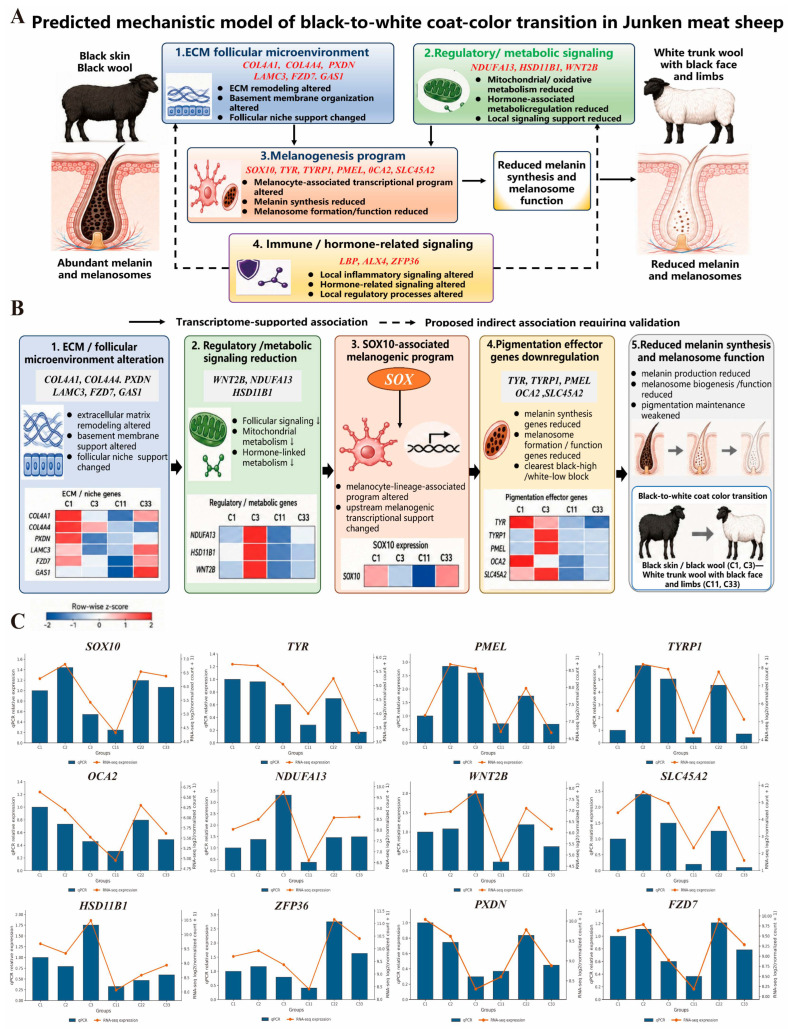
Hypothetical transcriptome-supported expression model of the follicular microenvironment–melanogenesis axis during the black-to-white coat-color transition in Junken meat sheep. (**A**) Integrated transcriptome-supported model showing coordinated transcriptional alterations in melanogenesis, ECM/follicular microenvironment, regulatory/metabolic signaling and immune/hormone-related signaling during black-to-white coat color transition. These modules were summarized based on differential expression analysis, functional enrichment, commonly downregulated gene module analysis and integrated candidate gene prioritization. (**B**) Predicted expression-associated model of core genes in the follicular microenvironment–melanogenesis axis and RNA-seq heatmap of representative genes. The proposed axis links ECM/follicular microenvironment alterations and regulatory/metabolic signaling changes with the *SOX10*-associated melanogenic program and the downregulation of pigmentation effector genes. Predicted relationships were inferred from coordinated RNA-seq expression patterns and require further validation. Heatmap values were calculated as row-wise z-scores after log2(FPKM + 1) transformation. Red indicates relatively high expression, and blue indicates relatively low expression. (**C**) RT-qPCR validation of 12 candidate genes using the same six biological samples as RNA-seq (three matched pairs: C1/C11, C2/C22, and C3/C33). Bars represent RT-qPCR relative expression levels, and orange lines indicate RNA-seq expression trends. Each sample was analyzed with three technical replicates. RT-qPCR was used to verify the consistency of expression trends between RNA-seq and selected candidate genes.

Based on RNA-seq expression patterns, functional enrichment results, and shared downregulated gene modules, we constructed a transcriptome-supported model of the black-to-white coat-color transition in Junken meat sheep (Figure 6). The most direct transcriptional feature of this model was the coordinated reduction in classical melanogenesis-related genes. Pigmentation-related genes, including *SOX10*, *TYR*, *TYRP1*, *PMEL*, *OCA2* and *SLC45A2*, were downregulated in white skin, suggesting attenuation of transcriptional programs associated with melanocyte lineage maintenance, melanin synthesis, and melanosome formation.

Beyond pigmentation effector genes, ECM/follicular microenvironment-related changes formed a second layer of the model. *COL4A1*, *COL4A4*, *PXDN*, *LAMC3*, *FZD7* and *GAS1* are associated with extracellular matrix composition, basement membrane structure, cell adhesion, and local signal transduction. Together with the recurrent enrichment of ECM–receptor interaction, extracellular region, focal adhesion, and basement membrane, these patterns suggest that changes in follicular structural support and the extracellular environment may provide a tissue-level context for reduced melanogenesis-related transcriptional activity. Regulatory and metabolic genes may further link follicular microenvironmental changes to pigmentation attenuation. *WNT2B*, *NDUFA13* and *HSD11B1* are related to local signaling, mitochondrial function, redox status, and hormone-associated metabolism, whereas *LBP*, *ALX4* and *ZFP36*, together with the Toll-like receptor, NF-kappa B, and estrogen signaling pathways, point to possible changes in immune-inflammatory, hormonal, and post-transcriptional regulatory environments. Because these signals showed greater sample-level heterogeneity than pigmentation effector genes, they may represent an auxiliary regulatory layer of follicular homeostasis rather than a single dominant regulatory factor.

Together, these findings support a transcriptome-based “follicular microenvironment–melanogenesis expression axis.” Changes in the ECM, follicular microenvironment, and regulatory/metabolic pathways were associated with lower expression of *SOX10*-related melanogenesis genes and pigmentation genes, including *TYR*, *TYRP1*, *PMEL*, *OCA2* and *SLC45A2*. This model should be regarded as an expression-based framework rather than evidence of a causal mechanism. Overall, the black-to-white coat-color transition in Junken meat sheep was accompanied by coordinated transcriptional changes in the follicular microenvironment, regulatory/metabolic pathways, and melanogenesis-related genes.

## 4. Discussion

The postnatal transition of Junken meat sheep from black fleece to a white-trunk phenotype represents a distinct developmental coat-color change, which may reflect attenuation of the hair follicle pigmentary unit during growth. In this study, RNA-seq analysis showed that classical melanogenesis-related genes were broadly downregulated in 179-day white-trunk-stage skin, accompanied by coordinated changes in ECM/follicular microenvironment-related signals, redox metabolism, Wnt/hormone-associated pathways, and immune-related processes. Together with previous omics and hair follicle biology studies, these findings suggest that the black-to-white coat-color transition is unlikely to be explained by a single pigmentation gene alone. Instead, it may involve multilayered transcriptional coordination among follicular microenvironmental remodeling, regulatory/metabolic signaling changes, attenuation of melanogenesis-related programs, and reduced expression of pigmentation effector genes.

The most direct transcriptional feature observed in this study was the overall decrease in pigmentation-related genes, including *SOX10*, *TYR*, *TYRP1*, *PMEL*, *OCA2* and *SLC45A2*, in white skin samples. This pattern is broadly consistent with previous omics studies of coat-color variation in small ruminants. Vasu et al. reported that *TYR*, *TYRP1*, *PMEL*, *OCA2* and *SLC45A2* were closely associated with dark skin pigmentation and coat-color differences in sheep transcriptomes [16,17]. Transcriptomic and histological analyses of Minxian black fur sheep by Shi et al. also showed that enhanced pigment deposition in black skin was accompanied by higher expression of melanogenesis-related genes [18]. At the proteomic level, Yin et al. further demonstrated coat-color-associated differences in proteins and functional pathways between black and white sheep skin [8]. In addition, studies in cashmere goats by Apar et al. and Wu et al. identified *TYR*, *TYRP1*, *DCT* and *PMEL* as important candidate genes involved in coat-color formation [19,20]. These studies, based on different omics layers, collectively support our finding that reduced expression of classical melanogenesis-related genes is one of the most stable and direct molecular features associated with coat-color fading in Junken meat sheep.

Mechanistically, these genes correspond to different key steps of the melanogenesis process. *TYR* encodes tyrosinase, the rate-limiting enzyme that catalyzes the initial reactions of melanin biosynthesis [19]. Thus, reduced *TYR* expression in white skin suggests a possible decrease in substrate conversion capacity for melanin synthesis. *TYRP1* is associated with eumelanin formation, melanogenic efficiency, and tyrosinase stability; its downregulation further supports the suppression of eumelanin synthesis in white skin [21,22]. *PMEL* is not simply a pigmentation enzyme but is involved in the formation of fibrillar scaffolds within premelanosomes, providing a structural platform for melanin deposition. Recent structural studies of the *PMEL* amyloid core and *PMEL* fibrils within melanosomes further demonstrate that PMEL-based fibrillar structures are essential for melanosome maturation and pigment deposition [23,24]. Therefore, the downregulation of *PMEL* indicates that white skin may not only exhibit reduced melanogenic enzyme activity but may also have impaired melanosome maturation and pigment-deposition platforms.

*OCA2* and *SLC45A2* are closely related to melanosomal pH, ionic homeostasis, and the biochemical environment required for melanogenic enzyme activity. Studies of melanosome transport and processing have shown that melanosomes are not passive pigment containers, but specialized organelles requiring finely regulated pH, ionic conditions, and protein trafficking [25,26,27]. Accordingly, downregulation of *OCA2* and *SLC45A2* may compromise the internal biochemical environment of melanosomes, thereby reducing the efficiency of melanin synthesis and deposition. These results suggest that the reduced expression of pigmentation-related genes in Junken meat sheep does not merely indicate lower expression of melanogenic enzymes, but instead points to coordinated attenuation across multiple levels, including melanocyte lineage status, melanogenic reactions, melanosome maturation, and the structural platform for pigment deposition.

Among the pigmentation-related candidates, the change in *SOX10* is particularly notable. Unlike pigmentation effector genes such as *TYR*, *TYRP1* and *PMEL*, *SOX10* is positioned closer to the upstream regulatory network governing melanocyte lineage maintenance and melanogenic programs. Cui and Man et al. highlighted that the development, migration, differentiation, and functional maintenance of mammalian melanocytes depend on the coordinated action of lineage-specific regulatory factors and local microenvironmental cues [28,29,30]. The concurrent downregulation of *SOX10* and multiple pigmentation effector genes in 179-day white-trunk-stage skin suggests broad attenuation of melanocyte-related transcriptional activity rather than suppression of a single pigmentation gene, consistent with dynamic melanocyte lineage states within the hair follicle niche [12]. Thus, the black-to-white coat-color transition in Junken meat sheep may reflect a developmental shift in the hair follicle pigmentary unit from an active melanogenic state toward a hypopigmented state.

Notably, ECM–receptor interaction, focal adhesion, extracellular region, basement membrane, and collagen-containing extracellular matrix repeatedly emerged across multiple analytical layers, and ECM/follicular microenvironment-related genes such as *COL4A1*, *COL4A4*, *PXDN*, *LAMC3*, *FZD7* and *GAS1* differed between black and white skin. Although these genes do not directly encode melanogenic enzymes, they may be important for interpreting developmental coat-color fading from the perspective of follicular architecture and pigmentary-unit maintenance.

The hair follicle is a highly organized mini-organ composed of epithelial cells, dermal mesenchymal cells, melanocytes, the basement membrane, and ECM. Melanocyte function is maintained within this niche and depends on both structural support and local signaling. Cell-ECM interactions are essential for hair follicle morphogenesis, cycling, and regeneration [31], and the specialized basement membrane helps maintain interactions between distinct tissue compartments [32]. Moreover, hair follicle epithelial stem cells can provide a functional niche for melanocyte stem cells, indicating that melanocyte-lineage maintenance is closely linked to the follicular microenvironment [33]. In this study, the recurrent enrichment of ECM- and adhesion-related terms, together with reduced expression of *COL4A1*, *COL4A4*, *PXDN*, *LAMC3*, *FZD7* and *GAS1* in white skin, suggests attenuation of the local follicular supportive environment. Given that ECM proteins and matrix mechanics can influence melanocyte differentiation, migration, and melanogenesis [34], these changes may influence reduced melanogenesis-related transcriptional activity during the black-to-white coat-color transition.

The downregulation of *COL4A1* and *COL4A4* may reflect changes in the perifollicular basement membrane and collagen scaffold, as collagen IV is a major basement membrane component required for tissue-interface stability [35,36]. Similarly, altered *LAMC3* and *PXDN* expression points to changes in ECM assembly and adhesion-related structure, because laminins contribute to basement membrane organization and cell adhesion [37,38], whereas *PXDN* promotes collagen IV network crosslinking and ECM stability [39]. Together with the enrichment of ECM–receptor interaction and focal adhesion, and consistent with previous findings linking these pathways to secondary hair follicle development in fine-wool sheep [7], our results suggest that ECM-related changes may provide a tissue-level explanation for the attenuation of pigmentation programs in white skin.

Among the ECM/microenvironment candidates, *FZD7* and *GAS1* may serve as signaling connectors. *FZD7* is a Frizzled receptor involved in Wnt signaling, and Frizzled genes show specific expression patterns in developing and postnatal hair follicles. Wnt receptors such as Fzd4 and Fzd7 are enriched in melanocyte stem cell-associated regions, suggesting a link between follicular niche signaling and melanocyte-lineage regulation [40,41]. Local Wnt, BMP, Shh, and Notch signals jointly regulate the hair follicle stem cell niche, hair cycling, and tissue homeostasis [35], while Wnt imbalance, oxidative stress, and inflammation are associated with follicle aging and pigmentation decline [42]. Thus, the altered expression of *FZD7* and *WNT2B* suggests that Wnt-related signaling may connect follicular microenvironmental changes with reduced melanogenesis. *GAS1*, a Hedgehog signaling-associated regulator, may also participate in local developmental signaling [43]. Although direct regulation of melanogenesis by *GAS1* was not demonstrated here, its coordinated change with ECM-related genes supports the possibility that local follicular cell communication is altered in white skin. Together, these findings suggest that changes in ECM and adhesion-related microenvironments may affect local Wnt/Hedgehog-associated signaling and thereby contribute to reduced melanocyte-lineage maintenance and melanogenesis-related transcriptional activity.

In this study, Module 1 was enriched for redox- and mitochondrial function-related terms, including oxidoreductase activity acting on NAD(P)H, NADH dehydrogenase activity, and respiratory chain complex I, with *NDUFA13* selected as a representative candidate gene. Melanogenesis is not only dependent on tyrosine metabolism and melanogenic enzyme activity, but is also influenced by cellular energy metabolism, redox balance, and mitochondrial function [4]. Mitochondrial redox regulation can affect tyrosinase stability, melanosome maturation, and eumelanin levels [44], while antioxidant systems are essential for melanocyte homeostasis and pigmentation [45]. *NDUFA13/GRIM-19*, a component of mitochondrial respiratory chain complex I, participates in complex I assembly, electron transport, and ROS-related regulation [46,47]. Therefore, the reduction in redox- and mitochondrial function-related modules in white skin, together with decreased expression of *TYR*, *TYRP1*, *PMEL*, *OCA2*, and *SLC45A2*, suggests a potential link between weakened metabolic support and reduced melanogenic efficiency during coat-color fading.

The altered expression of *HSD11B1* may reflect changes in the local hormonal or stress-related metabolic environment of the skin, as *HSD11B1* is expressed in skin and hair follicle-associated cells and can influence dermal papilla cell activity, keratinocyte proliferation, and skin homeostasis through glucocorticoid regulation [48,49]. Enrichment of arachidonic acid metabolism further suggests the involvement of lipid-mediated local regulation, since skin lipids and their bioactive metabolites are closely related to barrier function, immune signaling, and inflammatory regulation [50,51]. Immune-related signals should also be interpreted within the follicular microenvironmental context, as immune cells and immune-associated pathways contribute to hair follicle development, hair growth, and tissue homeostasis [52]. Likewise, PI3K-Akt and PI3K-Akt-mTOR signaling are involved in skin homeostasis, cell survival, immune inflammation, and barrier function [53,54]. Thus, changes in *LBP*, *ZFP36*, and TLR/NF-κB/IL-17-related signals may reflect adjustment of local follicular immune homeostasis or tissue state rather than direct core may influencers of coat-color fading.

This study has several limitations. Because the black-to-white coat-color transition in Junken meat sheep occurs during postnatal development, age, developmental stage, and coat-color phenotype are inherently linked; therefore, the transcriptomic differences observed between newborn black-fleece-stage skin and 179-day white-trunk-stage skin may reflect both coat-color transition and normal developmental processes, including skin maturation, hair follicle development, endocrine regulation, and immune maturation. In addition, the small sample size of three matched biological pairs limits statistical power and may increase the risk of false-positive or false-negative findings, so the identified DEGs should be regarded as candidate transcriptomic features rather than definitive population-level markers. Furthermore, this study was mainly based on RNA-seq analysis and RT-qPCR trend validation, without direct assessment of melanin deposition, melanocyte distribution, hair follicle morphology, protein expression, spatial localization, or cellular function. Therefore, the proposed follicular microenvironment–melanogenesis expression axis should be interpreted as a transcriptome-supported working model rather than a confirmed mechanism. Future studies using larger longitudinal cohorts, age-matched stable-color controls, additional developmental stages, histological and protein-level analyses, spatial validation, and functional experiments will be needed to verify and refine this model.

## 5. Conclusions

This study provides exploratory transcriptomic evidence that the natural postnatal black-to-white coat-color transition in Junken meat sheep is accompanied by coordinated changes in melanogenesis-related genes, ECM/follicular microenvironment-associated genes, and regulatory or metabolic pathways. The downregulation of *SOX10*, *TYR*, *TYRP1*, *PMEL*, *OCA2* and *SLC45A2* suggests attenuation of melanogenesis-related transcriptional activity, while changes in *COL4A1*, *COL4A4*, *PXDN*, *LAMC3*, *FZD7*, *GAS1*, *NDUFA13*, *WNT2B*, *HSD11B1*, *LBP*, *ALX4* and *ZFP36* indicate potential involvement of follicular niche, signaling, metabolic, and immune-related processes. Together, these findings support a transcriptome-based follicular microenvironment–melanogenesis expression axis as a working framework for understanding developmental coat-color fading in Junken meat sheep. Given the limited sample size and lack of functional validation, these candidate genes and pathways require further confirmation in larger cohorts and experimental models.

## Data Availability

The RNA-seq datasets generated and analysed during the current study are available in the NCBI Sequence Read Archive repository under BioProject accession number PRJNA1462536.

## Supplementary Materials

The following supporting information can be downloaded at: https://www.mdpi.com/article/10.3390/biology15131042/s1, Table S1. Sample information and matched longitudinal comparison design. Table S2: Primer information used for RT-qPCR; Table S3: Statistics of RNA-Seq data quality; Table S4: Statistics of reads aligned to genomic regions; Table S5: Statistics of reads aligned with the reference genome.
