# Supplementary material for: Transcriptome Analysis Reveals a Follicular Microenvironment Melanogenesis Axis in Black-to-White Coat-Color Transition of Junken Meat Sheep
Source: Biology (Basel). 2026 Jun 30;15(13):1042. doi: 10.3390/biology15131042 (PMC13360250; doi:10.3390/biology15131042)
Supplement: Supplementary file 1 [file biology-15-01042-s001.zip › biology-4345931-supplementary.pdf]

Transcriptome analysis reveals a follicular microenvironment melanogenesis axis in black to white coat color transition of Junken meat sheep

Supplementary Table S1. Primer information used for RT-qPCR.

Gene Name	Primer Sequences (5' - 3')	Product (bp)
SOX10	F: GCTGAATGAGAGCGACAAGC<br>R: CTTCCCGTTCTTCCGTCTCC	115
TYR	F: ACAACAGTCGCCAGGCTTTA<br>R: TCCTGGCTTTGTCGTGGTTT	81
TYRP1	F: AACACCTGCGACATTTGCAC<br>R: CGCAGACTACTCGCCATTGA	106
PMEL	F: CAGAAGGTACTGCAGGCTCC<br>R: GTCCAGGGTGAGGGAAAAGG	97
OCA2	F: CGTGCTCCTAAACCTGAGCA<br>R: GTTACCTCCCAGACAGGCAC	94
NDUFA13	F: TGTCGGGCTACAGCATGTTC<br>R: GGCCTCGAAGTCCTCAATCT	113
WNT2B	F: CCGGCGGCGAGCCTTAT<br>R: AGCATAGCTCCCCGAAGACT	120
SLC45A2	F: CCCAACCGGAGACCCTACATCCTCA<br>R: GATATGATCGCGTCCCCGTT	117
HSD11B1	F: TGCCTAGGAGGTTGTAGAAAGC<br>R: CCCAGAATGGGGAGGAGGTAT	115
ZFP36	F: GATCTCGCCGCCATCTACAA<br>R: GGACTCAGTCTCTCCGAGGT	75
PXDN	F: CGGGTCGTTTGAAGACTTGG<br>R: CGGGTCGTTTGAAGACTTGG	72
FZD7	F: GCACCATCATGAAGCACGAC<br>R: AGAAGTAGCAGGCCAGAACG	120
GADPH	F: CTGACCTGCCGCCTGGAGAAA<br>R: GTAGAAGAGTGAGTGTCGCTGTT	180

Supplementary Table S2. Statistics of RNA-Seq data quality.

Sample Name	Library Number	Raw Reads (bp)	Clean Reads (bp)	Error Rate	Q20	Q30	GC pct
C1	1	46546276	45422236	0.03	97.67	93.5	49.53
C2	2	46554704	45095028	0.03	97.58	93.38	50.42
C3	3	42443472	41369132	0.03	97.67	93.53	50.57
C11	4	47984356	46822620	0.03	97.72	93.52	45.51
C22	5	40722006	38723040	0.03	97.76	93.76	51.5
C33	6	46304822	45155682	0.03	97.57	93.19	50.06

Supplementary Table S3. Statistics of reads aligned to genomic regions.

Sample Name	Total Reads	Total Map	Unique Map	Multi Map
C1	45422236	39935990(87.92%)	37078208(81.63%)	2857782(6.29%)
C2	45095028	39239259(87.01%)	36464740(80.86%)	2774519(6.15%)
C3	41369132	36598853(88.47%)	33119690(80.06%)	3479163(8.41%)
C11	46822620	41713766(89.09%)	38080264(81.33%)	3633502(7.76%)
C22	38723040	33944023(87.66%)	31841093(82.23%)	2102930(5.43%)
C33	45155682	39811475(88.16%)	36500149(80.83%)	3311326(7.33%)

Supplementary Table S4. Statistics of reads aligned with the reference genome.

Sample Name	Exonic Region	Intronic Region	Intergenic Region
C1	4508529513(75.7102%)	739231796(12.4137%)	707216297(11.8761%)
C2	4406773450(75.3090%)	728475447(12.4492%)	716340918(12.2418%)
C3	4503820115(82.5033%)	330993796(6.0633%)	624137216(11.4333%)
C11	4455609268(71.6469%)	932919414(15.0015%)	830314729(13.3516%)
C22	3854074220(76.1236%)	654778977(12.9328%)	554064638(10.9436%)
C33	4675186906(78.7673%)	527475126(8.8869%)	732784373(12.3459%)

Supplementary Table S5. Sample information and matched longitudinal comparison design.

Individual ID	Sample ID	Age/developmental stage	Coat-color status at sampling	Sampling site	Stage grouping	Matched comparison
Individual 1	C1	Newborn / T0	Entirely black fleece at birth	Standardized dorsal trunk skin	Newborn black-fleece stage	C11 vs C1
Individual 1	C11	179 days / T179	White trunk fleece after postnatal development	Standardized dorsal trunk skin	179-day white-trunk stage	C11 vs C1
Individual 2	C2	Newborn / T0	Entirely black fleece at birth	Standardized dorsal trunk skin	Newborn black-fleece stage	C22 vs C2
Individual 2	C22	179 days / T179	White trunk fleece after postnatal development	Standardized dorsal trunk skin	179-day white-trunk stage	C22 vs C2
Individual 3	C3	Newborn / T0	Entirely black fleece at birth	Standardized dorsal trunk skin	Newborn black-fleece stage	C33 vs C3
Individual 3	C33	179 days / T179	White trunk fleece after postnatal development	Standardized dorsal trunk skin	179-day white-trunk stage	C33 vs C3
